# P-92. Risk factors, Clinical Manifestations and Management of Vertebral Osteomyelitis due to *Coccidioides*: a 10-year Retrospective Descriptive Study

**DOI:** 10.1093/ofid/ofae631.299

**Published:** 2025-01-29

**Authors:** Sona R Wolf, Fariba Donovan, Jacob Robishaw-Denton, Talha Riaz

**Affiliations:** University of Arizona College of Medicine Tucson, Tucson, Arizona; University of Arizona, Valley Fever Center for Excellence, Tucson, Arizona; University of Arizona College of Medicine Tucson, Tucson, Arizona; University of Arizona College of Medicine Tucson, Tucson, Arizona

## Abstract

**Background:**

Vertebral osteomyelitis is a severe manifestation of disseminated coccidioidomycosis with limited published data on clinical presentations and outcomes. The purpose of this study was to describe the epidemiology, risk factors, clinical features, management, and outcomes of coccidioidal vertebral osteomyelitis (CVO) among a cohort of patients in an endemic region.

**Methods:**

We conducted a retrospective chart review of patients treated for CVO between 2013 and 2022 at an academic health system in Arizona. Demographic and clinical data were extracted.

**Results:**

Forty-two patients with CVO were identified. The mean age at diagnosis was 36.3 (range, 18.9-73.2 years). Thirty-four (81%) patients were males and 33 (79%) were African American. Nine (21%) had a prior or current history of incarceration. Fifteen (36%) patients had a history of coccidioidomycosis including pulmonary (n=9), skin/musculoskeletal (n=5), and CNS infection (n=1) prior to diagnosis of CVO. Known comorbidities included long-term corticosteroid use (n=2), HIV infection (n=2), and pregnancy at time of CVO diagnosis (n=3 of 8 females). Of the 37 patients with complement fixation titers available at time of presentation, 25 patients had a titer >=1:128. The most frequent imaging findings identified via CT or MRI were paraspinal/epidural abscesses (n=32) or bone lytic lesions/erosions (n=32). All patients received triazole therapy, 32 (76%) received amphotericin B (median 15.5 days) at some point during their treatment course, and 4 (10%) received an investigational drug. Sixteen (38%) patients underwent surgical debridement and spinal fixation hardware was placed in 13 (31%). Thirteen (31%) patients experienced disease progression warranting re-admission. Four (10%) died within 3.5 years of diagnosis and all deaths were secondary to complications associated with CVO.

**Conclusion:**

There is a high morbidity and mortality due to CVO. Most patients with CVO are African American males and few have predisposing comorbidities. A remarkably high proportion (21%) have a history of incarceration suggesting it may be a risk factor for CVO. A better understanding of the factors that predispose individuals to CVO and better therapies are urgently needed to improve outcomes in this frequently devastating illness.

**Disclosures:**

**Fariba Donovan, MD, PhD**, F2G: Advisor/Consultant|F2G Ltd: Advisor/Consultant|F2G Ltd: Grant/Research Support|F2G Ltd: Honoraria

